# Fungi and Fungal Metabolites for the Improvement of Human and Animal Nutrition and Health

**DOI:** 10.3390/jof7040274

**Published:** 2021-04-04

**Authors:** Laurent Dufossé, Mireille Fouillaud, Yanis Caro

**Affiliations:** 1 Laboratoire de Chimie et Biotechnologie des Produits Naturels—CHEMBIOPRO, Université de la Réunion, 15 Avenue René Cassin, CEDEX 9, CS 92003, F-97744 Saint-Denis, Ile de la Réunion, France; mireille.fouillaud@univ-reunion.fr (M.F.); yanis.caro@univ-reunion.fr (Y.C.); 2 Ecole Supérieure d’Ingénieurs Réunion Océan Indien—ESIROI, 2 Rue Joseph Wetzell, F-97490 Sainte-Clotilde, Ile de la Réunion, France; 3 IUT de La Réunion, Département Hygiène, Sécurité, Environnement (HSE), 40 Avenue de Soweto, CEDEX 9, BP 373, F-97455 Saint-Pierre, Ile de la Réunion, France

**Keywords:** micro-fungi, macro-fungi, filamentous fungi, antioxidant, *Ganoderma*, kombucha, anticancer, carotenoid, medicinal mushroom, mycobiome, antimicrobial, antifungal, bioconversion, feed additive, cheese, dairy, *Sclerotinia*, secondary metabolite

Fungi: 1, 2, 3, ... 5.1 million species? Even scientists do not currently agree on how many fungi there might be on the planet Earth but only about 120,000 have been described so far [1]. They have been grouped into a separate kingdom of organisms, as complex and diverse as plants and animals, of which only a small percentage have been named and described. Fungal biomasses and fungal metabolites have a long common history with human and animal nutrition and health. Macro fungi and filamentous fungi bring a large portfolio of proteins, lipids, vitamins, minerals, oligo elements, pigments, colorants, bioactive compounds, antibiotics, pharmaceuticals, etc. For example, industrially important enzymes and microbial biomass proteins have been produced from fungi for more than 50 years. Some start-ups convert by-products and side-streams rich in carbohydrates into a protein-rich fungal biomass. The biomass is then processed into a vegan meat substitute for food applications. The last years there has also been a significant rise (in fact, a significant revival) in the number of publications in the international literature dealing with the production of lipids by microbial sources (the “single cell oils; SCOs” that are produced by the so-called “oleaginous” microorganisms, including “oleaginous” fungi, such as the zygomycete species, e.g., *Cunninghamella echinulata* and *Mortierella isabellina*). Fungi are potential sources of polyunsaturated fatty acids (PUFAs) as these microorganisms can accumulate large amounts of high-valued PUFAs, such as gamma-linolenic acid (GLA) and arachidonic acid (ARA).

The objective of the invitation to contribute to this MDPI Fungi Special Issue was not to give a complete coverage of how fungi and fungal metabolites are able to improve human and animal nutrition and health. We, as co-guest editors, simply wanted to encourage authors working in this field to publish their most recent work in a rapidly expanding journal, in order to make a large audience discover the full potential of wonderful and beneficial fungi. Thus, this Special Issue welcomes 22 scientific contributions (13 original research papers and 9 reviews) on applications of fungi and fungal metabolites, such as bioactive fatty acids, pigments, polysaccharides, alkaloids, terpenoids, etc., with great potential in human and animal nutrition and health.

Three original research papers investigate the advantages of using probiotics and living fungi in feed. Kanpiengjiai et al. [2] provide insights into a potential multifunctional probiotic yeast in aquaculture produced from *Sporidiobolus ruineniae* cultivated in tannin substrate. *Sporidiobolus ruineniae* A45.2, a carotenoid-producing yeast, was able to co-produce cell-associated tannase (CAT), gallic acid and viable cells with antioxidant activity when grown in a tannic acid substrate. Furthermore, viable cells were characterized by moderate hydrophobicity, high auto-aggregation and moderate co-aggregation with *Staphylococcus aureus*, *Salmonella ser. thyphimurium* and *Streptococcus agalactiae*. In another aspect of yeasts in animal feed, Keller et al. [3] investigate the gas-formation potential of yeasts present in liquid swine diets. Yeasts are said to cause sudden death in swine due to intestinal gas formation. As not all animals given high yeast content feed fall ill, growth and gas formation potential at body temperature were investigated as possible causally required properties. Most *Candida krusei* isolates formed high gas amounts within 24-h, whereas none of the *Candida lambica*, *Candida holmii* or most of the other isolates did. The gas pressure formed by yeast isolates varied more than tenfold. Only a minority of the yeasts were able to produce gas at temperatures common in the pig gut. Moving to dairy sheep milk performance, Mavrommatis et al. [4] evaluate the dietary administration of *Saccharomyces cerevisiae* live yeast on milk performance and composition, oxidative status of both blood plasma and milk, and gene expression related to the immune system of lactating ewes during the peripartum period. In conclusion, the dietary supplementation of ewes with *S. cerevisiae* improved the energy utilization and tended to enhance milk performance with simultaneous suppression of mRNA levels of pro-inflammatory genes during the peripartum period. Of course, fungi may also have an impact on human nutrition and health. Nguyen et al. [5] focus on *Monascus purpureus*, a filamentous fungus known for its fermentation of red yeast rice, that produces the metabolite monacolin K used in statin drugs to inhibit cholesterol biosynthesis. In their study, they show that active cultures of *M. purpureus* CBS 109.07, independent of secondary metabolites, use the mechanism of cholesterol assimilation to lower cholesterol in vitro. The findings demonstrate that active growing of *M. purpureus* CBS 109.07 can assimilate cholesterol, removing 36.38% of cholesterol after 48-h of incubation at 37 °C. The removal of cholesterol by resting or dead *M. purpureus* CBS 109.07 was not significant, with cholesterol reduction ranging from 2.75%–9.27% throughout a 72-h incubation. Cholesterol was also not shown to be catabolized as a carbon source.

The World Health Organization (WHO; Geneva, Switzerland) has established an urgent pathogen list of antibiotic-resistant bacteria to guide the research, discovery, and development of new antibiotics. This list includes carbapenem-resistant *Pseudomonas aeruginosa,* Enterobacteriaceae, and third generation cephalosporin-resistant bacteria as critical priorities as a result of the continuous and indiscriminate use of antibiotics, not only in the treatment of human diseases, but also in animals. This list includes antifungal compounds. Aguilar-Pérez et al. [6] explore and compare the antibacterial activity and chemical diversity of two endophytic fungi isolated from the plant *Hyptis dilatata* and cultured under different conditions by the addition of chemical elicitors, changes in the pH, and different incubation temperatures. A total of 34 extracts were obtained from both *Pestalotiopsis mangiferae* and *Pestalotiopsis microspora* and were tested against a panel of pathogenic bacteria. Three active extracts obtained from *P. mangiferae* were analyzed by Liquid Chromatography-Electrospray Ionization-Quadrupole-Time of Flight-Mass Spectrometry (LC-ESI-Q-TOF-MS) to screen the chemical diversity and the variations in composition. This allows the proposal of structures for some of the determined molecular formulas, including the previously reported mangiferaelactone, an antibacterial compound.

Arbuscular mycorrhizal fungi interact with plants, beneficial to humans or not. Habeeb et al. [7] highlight the integration of Arbuscular mycorrhizal fungi (AMF) and elevated CO_2_ (eCO_2_) into agricultural procedures as an ecofriendly approach to support the production and quality of plants. This study was conducted to investigate the effects of AMF and eCO_2_, individually or in combination, on growth, photosynthesis, metabolism and functional food value of *Thymus vulgare*. Results revealed that both AMF and eCO_2_ treatments improved photosynthesis and biomass production, however, much more positive impact was obtained by their synchronous application. Moreover, the levels of the majority of the detected sugars, organic acids, amino acids, unsaturated fatty acids, volatile compounds, phenolic acids and flavonoids were further improved as a result of the synergistic action of AMF and eCO_2_, as compared to the individual treatments.

Elicitation effects of fungal products on plants and mushrooms are screened all over the world. Kim et al. [8] investigate the elicitation effects of alginate oligosaccharides extracted from brown algae (*Sargassum* species) on β-glucan production in cauliflower mushroom (*Sparassis latifolia*). Sodium alginate was refined from *Sargassum fulvellum*, *S. fusiforme*, and *S. horneri*, and characterized by proton nuclear magnetic resonance spectroscopy. The Solid Fraction (SF) of *S. fusiforme* and Liquid Fraction (LF) of *S. horneri* were chosen for elicitation on *S. latifolia*, yielding the highest β-glucan contents of 56.01 ± 3.45% and 59.74 ± 4.49% in the stalk, respectively. Total polyphenol content (TPC), antioxidant activities (2,2′-Azino-bis (3-ethylbenzthiazoline-6-sulfonic acid) (ABTS) radical scavenging and Superoxide dismutase (SOD)-like activity) of aqueous extracts of *S. latifolia* were also greatly stimulated by alginate elicitation.

Vegetal biomass, available in large amounts on the planet, could be a source of fungal substrate to produce useful compounds for humans, animals or plants. The project conducted by Ibarra-Cantún et al. [9] determines the quantity of secondary metabolites and the antioxidant activity of the extracts obtained by the solid-state fermentation of apple and agave mezcalero bagasse over 28 days, inoculated with the *Pleurotus ostreatus* strain. Solid-state fermentation (SSF) is used in enzyme and antibiotic production, bioethanol and biodiesel as an alternative energy source, biosurfactants with environmental goals, and the production of organic acids and bioactive compounds. The results showed a higher presence of phenolic compounds, flavonoids, total triterpenes and antioxidant activity in the apple bagasse from the SSF on day 21 in the extract of acetone and water (80:20 *v/v*), 100% methanol and aqueous; while the agave bagasse showed a significant presence of phenolic compounds and flavonoids only in the aqueous extract. In conclusion, the presence of secondary metabolites exhibiting antioxidant activities from the solid-state fermentation in the residues of the cider and mezcal industry is an alternative use for wasted raw material, and reduces the pollution generated from agro-industrial residues.

Fungal biotechnology brings much progress to humans, animals, and plants. Five research papers illustrate this aspect by the production of lipids and pigments or colorants. The biomass of *Mucor circinelloides*, a dimorphic oleaginous filamentous fungus, has a significant nutritional value and can be used for single cell oil production. Metal ions are micronutrients supporting fungal growth and metabolic activity of cellular processes. Dzurendova et al. [10] study the effect of 140 different substrates, with varying amounts of metal and phosphate ions concentration, on the growth, cell chemistry, lipid accumulation, and lipid profile of *M. circinelloides*. It was observed that Mg and Zn ions were essential for the growth and metabolic activity of *M. circinelloides*. An increase in Fe ion concentration inhibited fungal growth, while higher concentrations of Cu, Co, and Zn ions enhanced the growth and lipid accumulation. Lack of Ca and Cu ions, as well as higher amounts of Zn and Mn ions, enhanced lipid accumulation in *M. circinelloides*. Generally, the fatty acid profile of *M. circinelloides* lipids was quite constant, irrespective of media composition. In a second study dedicated to lipids, *Mortierella alpina* was used by Slaný et al. [11] in SSF for the bioconversion of animal fat by-products into high value fermented bioproducts enriched with arachidonic acid (ARA). Although in general the addition of an animal fat by-product caused a gradual cessation of ARA yield in the obtained fermented bioproduct, the content of ARA in fungal biomass was higher. Thus, *M. alpina* CCF2861 effectively transformed exogenous fatty acids from animal fat substrate to ARA. Maximum yield of 32.1 mg of ARA/g of bioproduct was reached when using cornmeal mixed with 5% (*w/w*) of an animal fat by-product as substrate.

Fungal pigments and colorants tend to replace synthetic, chemical ones as consumers request green and sustainable processing. Demand for microbial colorants is now becoming a competitive research topic for food, cosmetics, textile, and pharmaceutics industries. Textile dyeing is one of the most polluting aspects of the global fashion industry, devastating the environment and posing health hazards to humans. Many fungal genera/species are currently in use for large-scale, industrial-scale production of pigments and colorants. *Talaromyces albobiverticillius* (deep red pigment producer), *Emericella purpurea* (red pigment producer), *Paecilomyces marquandii* (yellow pigment producer) and *Trichoderma harzianum* (yellow-brown pigment producer) are discussed by Lebeau et al. [12]. Polyketide-based pigments from fungal submerged cultures are usually extracted by conventional liquid–liquid extraction methods requiring large volumes of various organic solvents and time. To address this question from a different angle, the authors proposed an investigation the use of three different aqueous two-phase extraction systems using either ammonium- or imidazolium-based ionic liquids. Their findings led them to conclude that (i) these alternative extraction systems using ionic liquids as the means for greener extractant worked well for this extraction of colored molecules from the fermentation broths of the filamentous fungi investigated; (ii) tetrabutylammonium bromide, [N4444]Br-, showed the best pigment extraction ability, with a higher putative affinity for azaphilone red pigments; (iii) the back extraction and recovery of fungal pigments from ionic liquid phases remained the limiting point of the method under their selected conditions for potential industrial applications. In another study, de Oliveira et al. [13] produce yellow-orange-red colorants with *Talaromyces amestolkiae* in a stirred-tank bioreactor. The effect of the pH-shift control strategy from 4.5 to 8.0 after 96-h of cultivation is evaluated at 500 rpm, resulting in an improvement of natural colorant production, with this increase being more significant for the orange and red examples, both close to four-fold. Next, the fermented broth containing the colorants is applied to the preparation of cassava starch-based films in order to incorporate functional activity in biodegradable films for food packaging. The presence of fermented broth did not affect the water activity and total solids of biodegradable films as compared with the standard. In the end, the films are used to pack butter samples (for 45 days) showing excellent results regarding antioxidant activity. It is demonstrated that the presence of natural colorants is obtained by a biotechnology process, which can provide protection against oxidative action, as well as be a functional food additive in food packing biomaterials. The last original paper and the third on fungal pigments and colorants deals with pigments derived from spalting fungi that have previously shown promise as textile dyes; however, their use has required numerous organic solvents with human health implications. Palomino Agurto et al. [14] explore the possibility of using linseed oil as a carrier for the pigment from *Scytalidium cuboideum* as a textile dye. Colored linseed oil effectively dyed a range of fabrics, with natural fibers showing better coloration. Scanning electron microscopy (SEM) revealed a pigment film over the fabric surface. While mechanical testing showed no strength loss in treated fabric, colorfastness tests showed significant changes in color in response to laundering and bleach exposure with variable effects across fabric varieties. SEM investigation confirmed differences in pigmented oil layer loss and showed variation in pigment crystal formation between fabric varieties. Heating of the pigmented oil layer was found to result in a bright, shiny fabric surface, which may have potential for naturally weatherproof garments.

A special issue is also an opportunity to go deeper into some scientific subjects via reviews. The first of six published in this issue describes how fungal metabolism meets modern demands for healthy aging, functional foods, and sustainability. Takahashi et al. [15] remind us that aging-associated, non-transmissible chronic diseases (NTCD) such as cancer, dyslipidemia, and neurodegenerative disorders have been challenged through several strategies including the consumption of healthy foods and the development of new drugs for existing diseases. Consumer health consciousness is guiding market trends toward the development of additives and nutraceutical products of natural origin. Fungi produce several metabolites with bioactivity against NTCD as well as pigments, dyes, antioxidants, polysaccharides, and enzymes that can be explored as substitutes for synthetic food additives. Research in this area has increased the yields of metabolites for industrial applications through improving fermentation conditions, application of metabolic engineering techniques, and fungal genetic manipulation. Several modern hyphenated techniques have impressively increased the rate of research in this area, enabling the analysis of a large number of species and fermentative conditions. Their review thus focuses on summarizing the nutritional, pharmacological, and economic importance of fungi and their metabolites resulting from applications in the aforementioned areas, examples of modern techniques for optimizing the production of fungi and their metabolites, and methodologies for the identification and analysis of these compounds. 

The second review by Ye al. [16] focuses on selenium. Selenium (Se) is essential for human health, however, Se is deficient in soil in many places all around the world, resulting in human diseases, such as the notorious Keshan disease and Kashin–Beck disease. Therefore, Se biofortification is a popular approach to improve Se uptake and maintain human health. Beneficial microorganisms, including mycorrhizal and root endophytic fungi, dark septate fungi, and plant growth-promoting rhizobacteria (PGPRs), show multiple functions, especially increased plant nutrition uptake, growth and yield, and resistance to abiotic stresses. Such functions can be used for Se biofortification and increased growth and yield under drought and salt stress. The work summarizes the use of mycorrhizal fungi and PGPRs in Se biofortification, aiming to improving their practical use. 

Another way to protect humans from chemical hazards is to find biological and sustainable alternatives to chemical fungicides. Zhang et al. [17] highlight this specific point for controlling postharvest decay of fruit. Fruit plays an important role in human diet. Whereas fungal pathogens cause huge losses of fruit during storage and transportation, abuse of chemical fungicides leads to serious environmental pollution and endangers human health. Antagonistic yeasts (also known as biocontrol yeasts) are promising substitutes for chemical fungicides in the control of postharvest decay owing to their widespread distribution, antagonistic ability, environmentally friendly nature, and safety for humans. Over the past few decades, the biocontrol mechanisms of antagonistic yeasts have been extensively studied, such as nutrition and space competition, mycoparasitism, and induction of host resistance. Moreover, combination of antagonistic yeasts with other agents or treatments was developed to improve biocontrol efficacy. Several antagonistic yeasts are used commercially. In the review of Zhang et al. [17], the application of antagonistic yeasts for postharvest decay control is summarized, including the antagonistic yeast species and sources, antagonistic mechanisms, commercial applications, and efficacy improvement. Issues requiring further study are also discussed. 

In addition to the research papers dedicated to probiotics, two reviews illustrate the importance of this scientific field. Pais et al. [18] discuss how *Saccharomyces boulardii* became so successful. *Saccharomyces boulardii* is a probiotic yeast often used for the treatment of gastro-intestinal (GI) tract disorders such as diarrhea symptoms. It is genetically close to the model yeast *Saccharomyces cerevisiae* and its classification as a distinct species or a *S. cerevisiae* variant has long been discussed. Here, the authors review the main genetic divergencies between *S. boulardii* and *S. cerevisiae* as a strategy to uncover the ability to adapt to host physiological conditions by the probiotic. *S. boulardii*, which possesses discernible phenotypic traits and physiological properties that underlie its success as probiotic, such as optimal growth temperature, resistance to the gastric environment and viability at low pH. Its probiotic activity has been elucidated as a conjunction of multiple pathways, ranging from improvement of gut barrier function, pathogen competitive exclusion, production of antimicrobial peptides, immune modulation, and trophic effects. This work summarizes the participation of *S. boulardii* in these mechanisms and the multifactorial nature by which this yeast modulates the host microbiome and intestinal function. The second paper assigned to probiotics, written by Kunyeit et al. [19], summarize how probiotic yeasts can be applied against *Candida* species associated infections. Superficial and life-threatening invasive *Candida* infections are a major clinical challenge in hospitalized and immuno-compromised patients. Emerging drug-resistance among *Candida* species is exacerbated by the limited availability of antifungals and their associated side-effects. In the current review, authors discuss the application of probiotic yeasts as a potential alternative/combination therapy against *Candida* infections. Preclinical studies have identified several probiotic yeasts that effectively inhibit virulence of *Candida* species, including *Candida albicans*, *Candida tropicalis*, *Candida glabrata*, *Candida parapsilosis*, *Candida krusei* and *Candida auris*. However, *Saccharomyces cerevisiae* var. *boulardii* is the only probiotic yeast commercially available. In addition, clinical studies have further confirmed the in vitro and in vivo activity of the probiotic yeasts against *Candida* species. Probiotics use a variety of protective mechanisms, including posing a physical barrier, the ability to aggregate pathogens and to render them avirulent. Secreted metabolites such as short-chain fatty acids effectively inhibit the adhesion and morphological transition of Candida species. Overall, the probiotic yeasts could be a promising effective alternative or combination therapy for *Candida* infections.

Mushrooms have been valued as food and health supplements by humans for thousands of years. They are rich in dietary fiber, essential amino acids, minerals, and many bioactive compounds, especially those related to human immune system functions. Mushrooms contain diverse immunoregulatory compounds such as terpenes and terpenoids, lectins, fungal immunomodulatory proteins (FIPs) and polysaccharides. The distributions of these compounds differ among mushroom species and their potent immune modulation activities vary depending on their core structures and fraction composition chemical modifications. Zhao et al. [20] provide insight into the current status of clinical studies on immunomodulatory activities of mushrooms and mushroom products.

Mycotoxin is an important issue when dealing with human and animal health or nutrition. A specific outline by Chen et al. [21] discuss sphinganine-analog mycotoxins (SAMs). Sphinganine-analog mycotoxins (SAMs) include fumonisins from the *Fusarium* genus and *Alternaria alternata* f. sp. *lycopersici* (AAL) toxins. SAMs have shown diverse cytotoxicity and phytotoxicity, causing adverse impacts on plants, animals, and humans, and are a destructive force to crop production worldwide. The review summarizes the structural diversity of SAMs and encapsulates the relationships between their structures and biological activities. The toxicity of SAMs on plants and animals is mainly attributed to their inhibitory activity against the ceramide biosynthesis enzyme, influencing the sphingolipid metabolism and causing programmed cell death.

With the last articles, number 21 and 22 of this Journal of Fungi special issue, we return to fungal pigments. Fungi can produce a myriad of secondary metabolites, including pigments. Some of these pigments play a positive role in human welfare while others are detrimental. The review by Lin and Xu [22] summarizes the types and biosynthesis of fungal pigments, their relevance to human health, including their interactions with host immunity, and recent progress in studying their structure–activity relationships. Fungal pigments are grouped into carotenoids, melanin, polyketides, and azaphilones, etc. These pigments are phylogenetically broadly distributed. While the biosynthetic pathways for some fungal pigments are known, the majority remain to be elucidated.

Synthetic pigments/non-renewable coloring sources used normally in the textile industry release toxic substances into the environment, causing perilous ecological challenges. To be safer from such challenges, both academia and industry have explored the use of natural colorants such as microbial pigments. As explained by Venil et al. [23] such explorations have created a fervent interest among textile stakeholders in undertaking the dyeing of textile fabrics, especially with fungal pigments. The biodegradable and sustainable production of natural colorants from fungal sources is comparatively advantageous to synthetic dyes. The prospective scope of fungal pigments has emerged in the opening of many new avenues in textile colorants for wide ranging applications.

As a concluding remark, we, the Guest Editors, wish to thank all the authors and the reviewers for their significant contributions to this Special Issue and for making it a highly successful and timely collection of papers. Our acknowledgements also go to the whole MDPI team, i.e., assistant editors, editors, Editor-in-Chief, production office, website management, etc.
